# Spatial changes in leaf biochemical profile of two tea cultivars following cold storage under two different vapour pressure deficit (VPD) conditions

**DOI:** 10.1016/j.foodchem.2018.10.095

**Published:** 2019-03-30

**Authors:** Emma R. Collings, M. Carmen Alamar, Sally Redfern, Katherine Cools, Leon A. Terry

**Affiliations:** a Plant Science Laboratory, Cranfield University, Bedfordshire MK43 0AL, UK; b Unilever R&D Colworth, Sharnbrook, Bedfordshire MK44 1LQ, UK

**Keywords:** Catechins, Methylxanthines, Caffeine, Theobromine, Colour, Metabolic rate

## Abstract

Withering is considered a crucial stage of black tea processing. In this study, tea shoots from two cultivars (cvs. Yabukita and Clone 2) were stored at 5 °C, in either a low or high vapour pressure deficit (VPD) environment, to determine the impact of different withering rates on physiology (*viz*. respiration rate [RR], colour and moisture loss) and biochemical profile (*viz*. individual catechins, methylxanthines) of tea shoots (*Camellia sinensis*). Low VPD and high VPD conditions during withering increased caffeine levels in Clone 2 and Yabukita, respectively (*p* < 0.05). Caffeine levels steadily increased over time in both cultivars (*p* < 0.05), coinciding with a rapid decline in theobromine (TB). Furthermore, stems contained lower epigallocatechin gallate (EGCG) and caffeine (*ca*. 75 and 56%, respectively) compared to bud and larger leaf (LL) (*p* < 0.05). Overall, the results of this study highlight factors such as mechanical harvesting, and hard or soft withering, which could affect final tea beverage quality.

## Highlights

• Cold withering emphasised temporal changes in caffeine and theobromine content.
• Spatial variations in catechin content observed between bud, large leaf and stem.
• Vapour pressure deficit (VPD) conditions differentially affected caffeine levels.
• Implications of mechanically harvested tea on final quality are discussed.

## 1 Introduction

Tea, which is the most widely consumed beverage in the world after water, is produced from *Camellia sinensis* shoots consisting of two or three leaves, and an unopened terminal bud (Deb & Pou, 2016). The quality is defined by the appearance of dried tea leaves, and the colour, aroma and taste of the tea liquor (Zheng, Li, Xiang, & Liang, 2016). For black tea production, withering is considered the major processing step involving both physical and chemical changes (Deb & Pou, 2016). During withering, tea shoots are spread evenly on a fine meshed screen and ambient air is blown through for ca. 12–17 h, which encourages water loss and leaf softening (physical wither). The appropriate conditions for withering, such as temperature and relative humidity (RH), are not readily defined in the literature since they can vary depending on climate, producing region and the type of manufacturing process used (Tomlins & Mashingaidze, 1997). In addition to reducing the final moisture content in the leaf, withering allows various chemical changes to occur which are important for taste and flavour development (chemical wither) (Deb and Pou, 2016, Zhen, 2002). Despite the importance of withering, there is a paucity of work assessing and understanding the biochemical changes occurring in the plucked tea leaf. Furthermore, differences in the rate of withering by controlling vapour pressure deficit (VPD) conditions have not previously been explored.

In the tea plant, there are a variety of compounds which contribute towards the final quality of a tea beverage, which are reported to vary between cultivar and harvest season (Fang et al., 2017). Flavan-3-ols or flavanols are a subgroup of polyphenols that include catechins. Catechins are the major phenolic compounds present in tea representing 10–25% of the dry weight (DW) of a fresh green leaf (Kuhnert et al., 2010, Rani et al., 2011). The main catechin, accounting for 50–80% of the total catechin concentration, is (−)-epigallocatechin gallate (EGCG), followed by (−)-epigallocatechin (EGC), (−)-epicatechin (EC) and (−)-epicatechin gallate (ECG) (Sang et al., 2011, Song et al., 2012). During black tea production, most catechins are oxidised into theaflavins (TF) and thearubigins (TR), which contribute towards taste, brightness and colour of the tea liquor and consequently impact on quality (Ölmez & Yilmaz, 2010). Other compounds which can influence tea quality include gallic acid (GA) (biosynthesis precursor to gallate-type catechins) and caffeine, a major contributor to the bitter taste in tea (Song et al., 2012, Zhen, 2002). The chemical composition of brewed tea is very complex, and as such, it is important to gain insight into the chemical changes that occur during each stage of the tea process to enable producers to enhance and or maintain tea quality.

Cold storage has become an important factor in preserving postharvest quality of fresh horticultural produce (Deuchande and Carvalho, 2015, Falagán and Terry, 2018, Munhuweyi et al., 2015, Wills et al., 2007), and is ubiquitously used to store crops after harvest during periods of high yield and reduced processing capacity. In previous work, withering tea shoots down to a moisture content of 65–67% during 27 days of cold storage did not cause adverse effects to quality in comparison to traditional withering; as assessed by black tea brightness, catechins and caffeine (Ölmez & Yilmaz, 2010). In some producing countries, a cold wither is used to store or hold the leaf, followed by a short physical wither to reduce moisture (Tomlins & Mashingaidze, 1997). The influence of temperature on withering rate was assessed by Ghodake, Goswami, and Chakraverty (2006) where temperatures over 30 °C were detrimental to tea quality; while Owuor and Obanda (1996) described a general increase in catechins. However, neither study monitored the impact of different withering rates on leaf biochemical profile.

The aim of this research work was, therefore, to evaluate shoots from two cultivars, namely, Clone 2 (black tea variety) and Yabukita (green tea variety), stored under cold conditions (5 °C) and at either low or high VPD conditions to vary the rate of wither. Temporal changes in physiology and individual biochemical compounds were assessed for the whole shoot and across different spatial regions (viz. bud, large leaf [LL] and stem) to provide a better understanding of the changes that occur during the tea withering process.

## 2 Materials and methods

### 2.1 Plant material

Tea shoots from two varieties, Clone 2 (*C. sinensis* var. *assamica* [black tea variety]) and Yabukita (*C. sinensis* var. *sinensis* [green and black tea variety]), were harvested on the 4th July 2013 from the small tea plantation under glass in the UK based at Unilever R&D (Bedfordshire, UK). Samples were transported directly to Cranfield University (CU), UK, within 2 h of harvest. During transport, shoots were kept cool (9.5 ± 0.5 °C) to prevent excessive moisture loss. Potential changes in biochemistry from harvest to arrival at CU, were assessed on tea shoots from Clone 2 and Yabukita, which had been immediately snap-frozen at harvest.

### 2.2 Experimental design

At time zero (day 0), ethylene and colour measurements were performed on three replicate samples (4 shoots per sample) per cultivar, selected at random. The remaining shoots were evenly divided between 12 Lock & Lock™ (12 L) storage boxes (supplied by Wayfair, UK), with six boxes allocated per cultivar. Due to smaller leaf size, Yabukita were harvested as 3 leaves and a bud (3LB), while Clone 2 shoots were plucked as 2 leaves and a bud (2LB). Each sample box contained 45 tea shoots (2LB) and 90 shoots (3LB) for Clone 2 and Yabukita, respectively. Boxes were flushed continuously with air (200 mL min^−1^) using a blower manifold (custom built and supplied by Air Equipment, Beds., UK). Relative humidity inside the storage boxes was controlled by passing incurrent air through 100 mL glycerol solutions containing two different concentrations: [a] 90 and [b] 60% (v/v) glycerol (Fisher Scientific, Leicestershire, UK,) in distilled water to achieve 80–92% RH (high VPD) and 90–98% RH (low VPD) environments], respectively. RH was converted into VPD values using the formula described in Somboonkaew and Terry (2010). Shoots were stored under these conditions for 11 days at 5 °C.

**Fig. 1:**
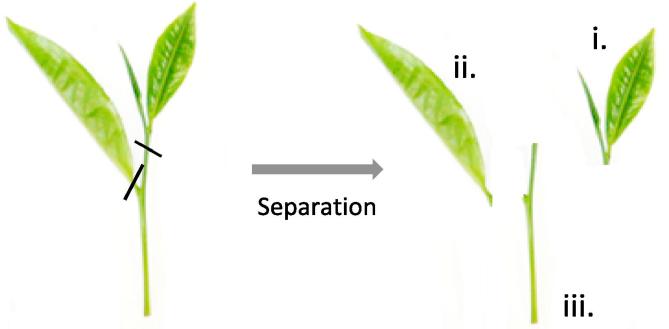
Schematic showing separation of shoots for spatial region (i. bud + small leaf, ii. large leaf [LL], iii. stem) assessment. Black solid lines represent where separation occurred.

At each sampling point (day 0, 1, 7 and 11), physiological assessments were performed (*viz*. respiration rate [RR], ethylene production and colour). After physiological measurements had been collected, samples were separated into three parts ([i] bud + small leaf, [ii] largest leaf and [iii] stem – see Fig. 1), snap-frozen in liquid nitrogen (BOC, Surrey, UK) and freeze dried at −50 °C. An additional time point at day 0 was also collected at Unilever R&D where samples were immediately snap frozen after harvest and used for further analysis. The individual weight (g) for each spatial region was recorded before and after freeze drying; this also enabled the calculation for percentage moisture loss. Dried samples were later used for biochemical analysis (*viz*. catechins and methylxanthines).

### 2.3 Physiological measurements

#### 2.3.1 Respiration rate and ethylene production

Respiration rate was measured directly from the storage boxes using a Sable Respirometry System (Model 1.3.8 Pro, Sable Systems International, Las Vegas, USA), which was calibrated with 10.06% CO_2_ and 1.99% O_2_ (10% CO_2_, 2% O_2_, 88% N_2_; certified standard from BOC, Surrey, UK) as described in Collings, García Cas, Ortiz, and Terry (2013) with slight modifications. Measurements were recorded using ‘push’ mode at a flow rate of *ca.* 100 mL min^−1^. Sample weight removed at each sampling point was recorded to adjust for RR calculations.

Ethylene production was analysed using an ETD-300 Ethylene Detector with a CAT-1 catalyser and VC-1 valve control box (Sensor Sense, Netherlands). Samples (n = 9 and 18 shoots for Clone 2 and Yabukita, respectively) were placed into cuvettes (250 mL) which had a continuous flow (4 L h^−1^) of hydrocarbon free air flushing through (Collings, Gavidia, Cools, Redfern, & Terry, 2018). A stable baseline measurement, obtained using an empty cuvette, was recorded at the beginning and end of each run to allow values to be adjusted by removing the baseline. Sample cuvettes were measured for *ca.* 6 to 10 min to allow a stable measurement to be recorded.

#### 2.3.2 Colour

Objective colour (lightness (L*), chroma (C*) and hue angle (h°) were measured using a Minolta CR-400 colourimeter with an 8 mm light aperture and DP-400 data processor (Minolta Co. Ltd., Japan) (Collings et al., 2018). The mean was calculated based on three readings measured from the largest leaf.

### 2.4 Biochemical analysis

Catechins and methylxanthines were extracted and quantified according to ISO 14502-2:2005. Two consecutive 70% (v/v) hot methanol (70 °C) extractions (2.5 mL followed by 2 mL) were performed on 200 mg DW of finely ground tea leaf sample (particle size between 20 and 30 µm). Samples were vortexed, placed in a water bath at 70 °C for 10 min and centrifuged at 875*g*-force for 5 min. The extract was decanted into a 5 mL volumetric flask. Both extracts were combined and 500 µL of leaf stabilising solution (2.5 mg mL^−1^ ethylenediaminetetraacetic acid (EDTA) and 2.5 mg mL^−1^ ascorbic acid in distilled water) was added before making up to 5 mL volume with cool aqueous methanol (70%). The final extract was filtered through a 0.2 µm PTFE syringe filter and analysed using an Agilent 1200 series HPLC system (Agilent, Berks., UK) with diode array detection (HPLC-DAD).

Caffeine standards of known concentrations and undiluted samples were injected (10 µL) into an Agilent ZORBAX Eclipse plus phenyl hexyl (4.6 mm × 250 mm, 5 µm particle size, Part no. 959990-912) fitted with an Agilent ZORBAX Eclipse phenyl hexyl XDB-C18 guard column (4.6 mm × 12.5 mm, 5 µm particle size, Part no. 820950-938). The mobile phase consisted of 2% acetic acid (Fisher Scientific, UK) in acetonitrile (Fisher Scientific, UK) (A) and 2% acetic acid + 20 mg mL^−1^ EDTA (Sigma Aldrich, Dorset, UK) in water (B). Separation was achieved using a gradient with a linear decrease/increase of solvent B: 100–95%, 10 min; 95%, hold 10 min; 95–90%, 5 min; 90–88.5%, 5 min; 88.5%, hold 28 min; 88.5–0%, 1 min; 0%, hold 11 min; 0–100%, 1 min; plus a post run of 100%, 7 min at a flow rate of 1 mL min^−1^ and a column temperature of 30 °C. Compounds were detected using an Agilent 1200 series HPLC-DAD (Model G1315D) set to a wavelength of 278 nm. Peak identification was achieved by comparison with known standards (gallic acid [GA], theobromine [TB], (+)-catechin, epicatechin [EC], epigallocatechin [EGC], epigallocatechin gallate [EGCG], epicatechin gallate [ECG], and caffeine) purchased from Sigma-Aldrich (Dorset, UK); and quantification was performed using relative response factors to caffeine. The limit of detection (LOD) and limit of quantification (LOQ) for each compound was determined as <0.005 and <0.02 mg mL, respectively.

### 2.5 Statistical analysis

Statistical analyses were carried out using Statistica for Windows version 10, 64-bit (Statsoft Tulsa, OK 74104, USA). Analysis of variance (ANOVA) was used to identify any significant differences (*p* < 0.05) between treatments followed by Tukey’s post-hoc test. Where VPD treatments were not found to be significant (*p* > 0.05), datasets were statistically re-assessed using only high VPD data and graphs/tables were plotted, accordingly. Furthermore, the standard deviation (SD) for each mean is displayed in each applicable Fig. and Table, and represents the standard error estimated from the residual mean square (Webster, 2007).

## 3 Results

### 3.1 Metabolic rate of fresh tea shoots during storage

**Fig. 2:**
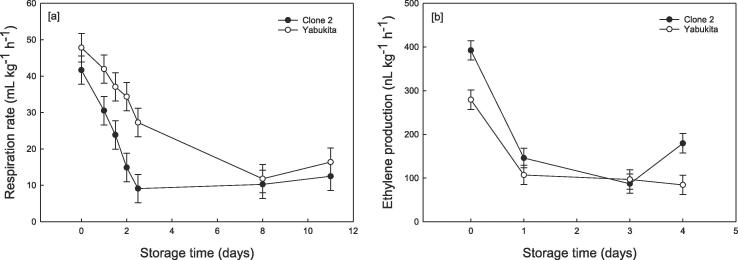
Changes in [a] respiration rate as CO_2_ produced (mL kg^−1^h^−1^) and [b] ethylene production (nL kg^−1^h^−1^) of two varieties of tea (*viz.* Clone 2 and Yabukita) during 11 days storage at 5 °C and high VPD. Ethylene data only shown up to day 4 due to falling below LOQ. Data represent means (*n* = 3) ± standard deviation (SD).

During 11 days of storage at 5 °C, RR in both tea varieties (cv. Clone 2 and Yabukita) significantly decreased from *ca.* 50 to 10 mL CO_2_ kg^−1^h^−1^ (Fig. 2a). Differences between cultivars were observed whereby Yabukita shoots had significantly higher overall mean values for RR (32.18 ± 1.47 mL CO_2_ kg^−1^h^−1^) compared to Clone 2 (21.59 ± 1.47 mL CO_2_ kg^−1^h^−1^) when stored under high VPD. However, this was not evident between cultivars under a low VPD environment.

For both cultivars, endogenous ethylene levels were relatively low with values decreasing during storage (Fig. 2b). Ethylene production as an overall mean value during 4 days storage at 5 °C was significantly higher in Clone 2 (201.24 ± 11.12 nL kg^−1^h^−1^) compared to Yabukita (141.87 ± 11.12 nL kg^−1^h^−1^). Ethylene measurements were not collected after 4 days as values dropped below the limit of quantification. No significant differences in RR or ethylene production were observed between the two VPD treatments over storage time (data not shown).

### 3.2 Temporal changes in tea shoot DW (%) and colour under different VPD conditions

Shoots stored under high VPD conditions had significantly higher DW (%) as a proportion of fresh weight (FW) compared to samples stored under low VPD, irrespective of cultivar (Table 1). Differences in DW were significant only towards the end of storage (day 7 and 11).

**Table 1:** Changes in dry weight (DW) (%) as a proportion of FW of tea shoots (cultivars combined) during 11 days cold storage (5 °C) under low and high vapour pressure deficit (VPD). Baseline sampling (B1) was taken at Unilever R&D. Data represent means (*n* = 60) ± standard deviation (SD). Different subscript letters denote significant differences.

Sampling day	DW (%)	
	Low VPD	High VPD
B1	21.08 ± 1.21^ab^	21.08 ± 1.21^ab^
1	20.15 ± 1.21^ab^	20.37 ± 1.21^ab^
4	18.79 ± 1.24^a^	19.55 ± 1.21^ab^
7	21.52 ± 1.21^ab^	24.45 ± 1.21^b^
11	22.99 ± 1.21^ab^	32.15 ± 1.21^c^

During 11 days storage at 5 °C, a gradual change in colour from green to dark green was observed as indicated by increasing h° from *ca*. 120 to 126 (Fig. S1). However, this trend was more rapid for Clone 2 (*C. assamica* variety) which had significantly higher h° values on day four (125.50 ± 0.79) compared to Yabukita (*C. sinensis* variety) (121.57 ± 0.79), coinciding with lower L* (35.42 ± 1.02 vs. 41.54 ± 1.02) and C* (19.65 ± 1.30 vs. 29.26 ± 1.30), respectively. No significant differences in leaf colour were shown between the two VPD treatments.

### 3.3 Spatial and temporal changes in tea shoot methylxanthines

Caffeine was the only compound found to be affected by VPD conditions during storage but trends varied between the two cultivars. Maintaining shoots under a low VPD environment was found to overall significantly increase caffeine concentrations (43.19 ± 0.37 mg g^−1^) compared to high VPD (41.67 ± 0.38 mg g^−1^) in Clone 2 shoots; whereas a contrasting effect was found in Yabukita with caffeine observed at 27.44 ± 0.37 and 28.81 ± 0.37 mg g^−1^ DW for low VPD and high VPD, respectively. Interactions between sampling day × VPD × spatial region were not significant for caffeine or TB in either cultivar (Tables S1 and S2).

**Fig. 3:**
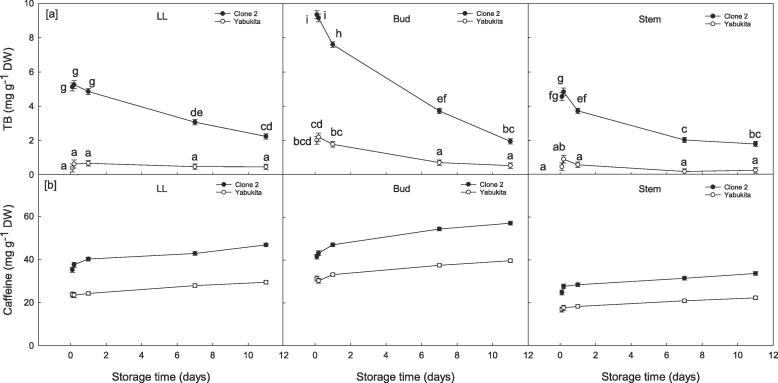
Spatial changes in [a] theobromine (TB) and [b] caffeine in fresh tea shoots (large leaf [LL], bud and stem) from two varieties of tea (*viz.* Clone 2 and Yabukita) during 11 days storage at 5 °C and under high vapour pressure deficit (VPD). Data represent means (*n* = 3) ± standard deviation (SD). Different letters denote significant differences.

At the start of storage, significant differences in TB were noted between the two cultivars, where Clone 2 had up to *ca*. 4.5-fold higher levels compared to Yabukita (Fig. 3a) under high VPD. During cold storage, both cultivars experienced a decrease in TB but this drop was more pronounced for Clone 2 with levels falling from *ca*. 9 mg g^−1^ DW to *ca*. 2 mg g^−1^ DW. In parallel to the decrease in TB, caffeine levels in Clone 2 and Yabukita significantly increased during storage (from *ca*. 33.84 ± 0.65 vs. 23.93 ± 0.65 mg g^−1^ DW to 45.85 ± 0.46 vs. 30.43 ± 0.46 mg g^−1^ DW) with Clone 2 having the highest concentrations, respectively. The interaction between sampling day × cultivar × spatial (Fig. 3b) was not significant for caffeine, but the graph is shown to provide a comparison between trends for caffeine and TB during storage. By the end of storage, all spatial regions contained similar TB levels, but concentrations remained higher in Clone 2 (Fig. 3a).

Spatial differences in caffeine and TB were noted where concentrations were overall lower in the stem (29.18 ± 0.42 vs. 19.17 ± 0.42 mg g^−1^ DW) compared to the bud (48.7 ± 0.42 vs. 34.38 ± 0.42 mg g^−1^ DW) followed by the LL (40.48 ± 0.43 vs. 25.71 ± 0.42 mg g^−1^ DW) in Clone 2 and Yabukita, respectively.

### 3.4 Temporal and spatial changes in tea shoot catechins

**Fig. 4:**
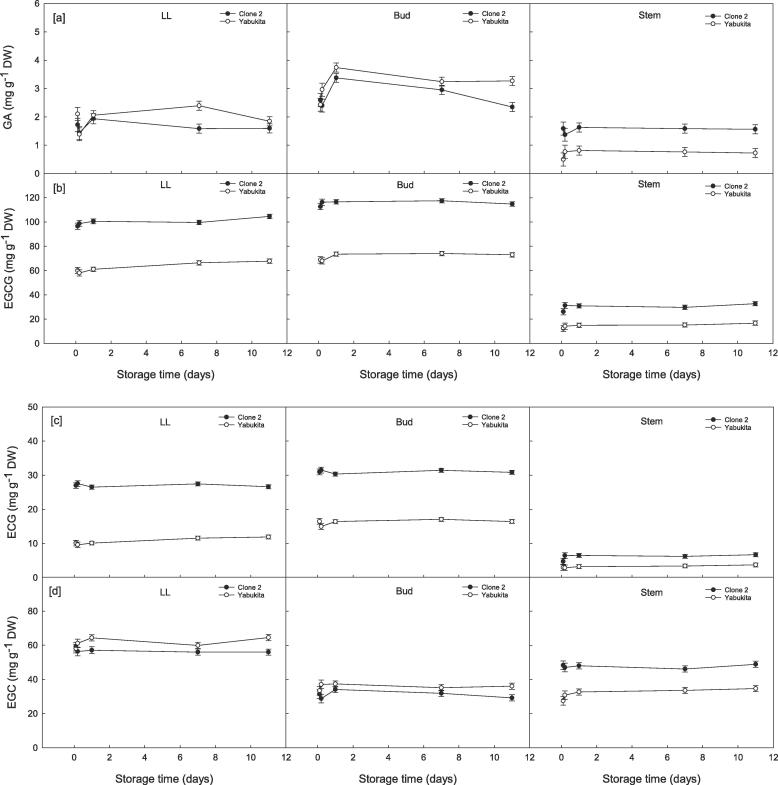
Spatial changes in [a] gallic acid (GA), [b] epigallocatechin gallate (EGCG), [c] epicatechin gallate (ECG), [d] epigallocatechin (EGC) (mg g^−1^ dry weight [DW]) in fresh tea shoots (large leaf [LL], bud and stem) from two varieties of tea (Clone 2 and Yabukita) during 11 days storage at 5 °C and under high vapour pressure deficit (VPD). Data represent means (*n* = 3) ± standard deviation (SD).

During cold storage, catechin levels remained fairly stable with no significant differences noted between VPD conditions (Tables S1 & S2). However, variations were evident between cultivars with Clone 2 generally containing the highest catechin levels (*viz.* [+]-catechin, EC, EGCG, ECG, EGC) in comparison to Yabukita (Figs. 4 and S2). For the main catechin EGCG, concentrations were 1.6-fold higher in Clone 2 (Fig. 4b).

Separation of the three spatial sections from a tea shoot (large leaf [LL], bud and stem) also revealed spatial differences in catechin content. For both tea cultivars, GA, EGCG and ECG in the stem regions were overall significantly lower compared to the bud and large leaf (Fig. 4a, b and d). Contrastingly, EGC and (+)-catechin levels in Clone 2 shoots only, were higher in the stem (47.67 ± 0.95 and 4.25 ± 0.12 mg g^−1^ DW) compared to the bud (31.10 ± 0.95 and 2.30 ± 0.11 mg g^−1^ DW), respectively (Figs. 4c and S2).

For the majority of catechins assessed, no significant differences in concentration were observed between samples snap-frozen at harvest and upon arrival at the laboratory. The only exception was for (+)-catechin in Clone 2 shoots which fluctuated in the stem and bud (Table S1).

## 4 Discussion

### 4.1 Temporal changes in tea shoot physiology

Physical withering is typically regulated at a slow rate for a long period to ensure adequate chemical withering. This is usually achieved by regulating the airflow and temperature (Deb & Pou, 2016), but in this experiment, leaves were exposed to different RH conditions at a constant temperature. Consequently, by varying the concentrations of glycerol, different rates of withering were achieved, as indicated by variations in DW (%) recorded for the two VPD conditions described. Despite this, the impact of VPD on the tea physiology was minimal.

Changes to leaf physiology and biochemistry during withering, such as chlorophyll breakdown (*ca*. 15%), are important for black tea appearance. Insufficient withering has been linked to the green off-colour in manufactured tea (Deb & Pou, 2016). Hue angle did not significantly change in Yabukita shoots (*C. sinensis* var. *sinensis*; green tea variety) but cold storage was found to darken tea leaves more rapidly in Clone 2 (*C. sinensis* var. *assamica;* black tea variety) shoots. Previous literature has reported darkening of spinach leaves stored for long periods (up to 16 days at 10 °C) (Hodges & Forney, 2000). This colour change is associated with chlorophyll degradation, resulting in the formation of pheophytin and chlorophyllide, and enzymatic oxidation of polyphenols, such as catechins, into red/brown compounds called TF and TR (Sabhapondit et al., 2012, Sang et al., 2011). That said, catechin levels remained stable during storage (see discussion below), but it is possible that some oxidation may have occurred concurrently with catechin synthesis. Leaf darkening in Clone 2 also coincided with higher ethylene production. Ethylene associated changes in colour of other leafy produce usually hastens chlorophyll loss and a gradual yellowing.

Although shoots are often processed soon after harvest, research related to the metabolic rate of tea shoots is scant. To the best of our knowledge, this is the first study to report changes in tea shoot RR during the withering process. Respiration rate was only significantly higher in Yabukita shoots under high VPD conditions, indicating that metabolic substrates such as non-structural carbohydrates may have depleted more rapidly, which could have implications for flavour. Previous literature reports that carbohydrates are converted into more simple sugars which then react with amino acids to produce black tea flavour compounds (Deb and Pou, 2016, Zheng et al., 2016). Furthermore, volatile composition has been shown to be affected by storage temperature. Increased volatiles with desirable floral, fruity and sweet flavours were detected in fresh green tea leaves stored at low temperatures (15 °C) in comparison to ambient conditions (25 °C) (Katsuno et al., 2014). Consequently, specific VPD conditions and cold storage could impact the volatile profile of final tea extract, although this was not assessed herein.

### 4.2 Spatial differences in biochemical content within a tea shoot

Detailed studies on the ratio of individual catechins in specific spatial regions of a tea shoot have previously been reported; however, most researchers report total catechin concentrations. Furthermore, there are a limited number of studies on biochemical differences within a stem which also contributes towards final tea flavour. In this study, many of the individual catechins tested had significantly different concentrations in the stem compared to the bud, and this was also observed for the methylxanthines. The main catechin, EGCG, was 75% lower in the stem compared to the bud and LL in Clone 2 (black tea cultivar); while caffeine levels were significantly lower in the stem of both cultivars. Differences in chemical composition between the stem and leaf/bud material could potentially affect the final quality of tea if more stem is incorporated into the final tea product. With ever increasing labour shortages, many tea plantations are relying on mechanical harvesting. Final tea beverages produced from mechanically harvested material have been shown to contain 1.3-times more stem material compared to hand plucked tea (Madamombe, Tesfamariam, & Taylor, 2015). Differences in chemical (volatile) composition were also reported in stem material used to produce oolong tea (semi-fermented) but this was found to have minimal effect on flavour (Zeng, et al.. , 2017). Variations in the level of catechins or methylxanthines in the different shoot material has been reported by Li et al. (2016), where catechin and caffeine levels were higher in younger leaves compared to mature leaves; however, stem material was not assessed. Differences in catechin content could potentially influence the formation of TF and TR, which significantly contribute towards briskness, brightness, strength and colour in black tea (Sabhapondit, Bhattacharyya, Bhuyan, Hazarika, & Goswami, 2014). Furthermore, teas with low levels of caffeine are considered poor quality due to insufficient creaming properties (Deb and Pou, 2016). Additional work would therefore be required to confirm the effects of mechanical harvesting on final tea quality.

### 4.3 Biochemical profile of tea shoots during cold wither

Some of the compounds known to be altered during withering include catechins and caffeine, where levels decrease and increase, respectively (Deb and Pou, 2016). The decrease in catechins was also observed by Ölmez and Yilmaz (2010) during cold withering; however, for Clone 2 and Yabukita shoots, cold storage retained similar levels of catechins throughout storage. It is possible that oxidation of catechins into TF was inhibited, since final moisture content in a withered leaf is known to have a significant inhibitory effect on enzyme activity (mainly polyphenol oxidase [PPO]) responsible for the formation of TF and TR (Sabhapondit et al., 2014). That said, other chemical changes were still evident within the Clone 2 and Yabukita shoots, including the observed increase in caffeine (up to *ca.* 28.3 and 35.5% for Yabukita and Clone 2, respectively) and decrease in TB. Alternatively, electron microscopy work has shown that catechins are localised within vacuoles (Suzuki, Yamazaki, Sada, Oguni, & Moriyasu, 2003); therefore, intracellular compartmentation of catechins may have prevented enzyme oxidation, but additional work would be required to confirm this.

Temporal changes in caffeine during withering have previously been reported in tea (Ölmez & Yilmaz, 2010), but this has not been the case for TB. Theobromine (caffeine precursor) concentrations in Clone 2 and Yabukita rapidly dropped during storage in all spatial regions (from day 0 to day 11, average values for Clone 2 and Yabukita buds changed from 9.2 to 2.0 mg g^−1^, and from 2.1 to 0.5 mg g^−1^, respectively). The rate of TB decline (up to 77% in Clone 2 buds) was 2-fold greater than the increase in caffeine (up to 38% in Clone 2 buds) on a percentage basis. The amount of caffeine generated (*ca*. 15 mg g^−1^ in Clone 2 buds) is consistent with all the depleted TB (*ca*. 7 mg g^−1^ in Clone 2 buds) being converted to caffeine. Interestingly, a similar increase in caffeine levels (*ca*. 29%) was observed in Yabukita, despite this clone having much lower levels of the precursor, TB.

Chemical withering occurs immediately after plucking and is considered independent of the rate of moisture loss, but instead is a function of temperature and time (Deb & Pou, 2016). In this study, withering under low and high VPD conditions (replicating soft and hard withering conditions, respectively) was found to influence caffeine levels but the trend was different between the two cultivars. In Yabukita shoots, a hard wither resulted in significantly higher caffeine levels compared to Clone 2, which also coincided with higher RR and DW (%). In contrast, a softer wither was more beneficial for increasing caffeine concentrations in Clone 2 shoots, but unlike Yabukita, different VPD conditions did not alter metabolic rate. It is postulated that accumulation of caffeine in young leaves acts as a chemical defence protecting young soft tissue against pathogens and herbivores (Ashihara, Kato, & Crozier, 2011). Consequently, the higher RR in Yabukita shoots under high VPD (hard wither) may have been a stress response triggering the accumulation of caffeine. There is likely to be significant variation in trends between the two cultivars due to varietal differences; Clone 2 (*C. sinensis* var. *assamica*) is typically used for black tea production, while Yabukita is used also for green tea production (*C. sinensis* var. *sinensis*). Despite observing differences in biochemistry following different withers, careful consideration is still required to ensure shoots have reached adequate moisture levels for subsequent processing (rolling or maceration) (Sabhapondit et al., 2014, Tomlins and Mashingaidze, 1997). Applying controlled VPD conditions, according to specific cultivar requirements could potentially enhance caffeine levels within tea extracts (particularly in varieties with low caffeine content) to improve tea creaming properties and thus quality.

## 5 Conclusion

Cold storage of fresh tea shoots stored under different VPD conditions was shown to have a differential effect on RR depending on cultivar. In addition, VPD influenced caffeine content where a hard (high VPD) and soft (low VPD) wither may be beneficial for Yabukita and Clone 2 shoots to increase caffeine content, respectively. Furthermore, spatial variations in catechin content, particularly with respect to the stem, could have a significant impact on tea beverage quality produced from mechanically harvested shoots. This study can help support crop management decisions enabling farmers to maintain tea quality.
