# Supplementary material for: Spatial changes in leaf biochemical profile of two tea cultivars following cold storage under two different vapour pressure deficit (VPD) conditions
Source: Food Chem. 2019 Mar 30;277:179–85. doi: 10.1016/j.foodchem.2018.10.095 (PMC6283014; doi:10.1016/j.foodchem.2018.10.095)
Supplement: Supplementary data 1 [file mmc1.docx]

**Supplementary data for paper entitled ‘Spatial changes in leaf biochemical profile of two tea cultivars following cold storage under two different vapour pressure deficit (VPD) conditions’.**

**Fig. S1.** Changes in colour (lightness [L*], chroma [C*] and hue angle [h°]) of two varieties of tea (*viz.* Clone 2 and Yabukita) during 11 days storage at 5 ^o^C under high vapour pressure deficit (VPD). Data represent means (*n* = 6) ± standard deviation (SD). Different letters denote significant differences.

**Table S1.** Spatial changes in gallic acid (GA), (+)-catechin, epicatechin (EC), epigallocatechin gallate (EGCG), epicatechin gallate (ECG), epigallocatechin (EGC), theobromine (TB) and caffeine (mg g^-1^ dry weight [DW]) in fresh tea shoots (large leaf [LL], bud and stem) from one variety of tea (Clone 2) during 11 days storage at 5^o^C and high [90% glycerol] and low [60% glycerol] vapour pressure deficit (VPD). Data represent means (n = 3) ± standard deviation (SD). Interactions between main factors studied (storage time x VPD x spatial) were not significant (*p* >0.05).

| **Sampling day** | **VPD** | **Glycerol (%)** | **Spatial** | **GA** | **EGCG** | **EGC** | **(+)-catechin** | **EC** | **ECG** | **Caffeine** | **TB** |
| --- | --- | --- | --- | --- | --- | --- | --- | --- | --- | --- | --- |
| B1 | * | * | LL | 1.72 ± 0.23 | 96.49 ± 3.18 | 59.20 ± 2.50 | 2.26 ± 0.49 | 23.18 ± 0.89 | 26.98 ± 0.95 | 35.20 ± 1.12 | 5.13 ± 0.31 |
| B1 | * | * | Bud | 2.59 ± 0.23 | 112.71 ± 3.18 | 31.41 ± 2.50 | 1.87 ± 0.49 | 18.36 ± 0.89 | 30.99 ± 0.95 | 41.57 ± 1.12 | 9.34 ± 0.31 |
| B1 | * | * | Stem | 1.59 ± 0.23 | 26.09 ± 3.18 | 48.26 ± 2.50 | 4.43 ± 0.49 | 21.00 ± 0.89 | 4.72 ± 0.95 | 24.76 ± 1.12 | 4.57 ± 0.31 |
| B2 | * | * | LL | 1.44 ± 0.23 | 98.65 ± 3.18 | 56.37 ± 2.50 | 2.19 ± 0.49 | 21.66 ± 0.89 | 27.57 ± 0.95 | 37.50 ± 1.12 | 5.26 ± 0.31 |
| B2 | * | * | Bud | 2.40 ± 0.23 | 116.27 ± 3.18 | 28.81 ± 2.50 | 3.20 ± 0.49 | 17.60 ± 0.89 | 31.50 ± 0.95 | 43.16 ± 1.12 | 9.15 ± 0.31 |
| B2 | * | * | Stem | 1.37 ± 0.23 | 31.25 ± 3.18 | 47.05 ± 2.50 | 3.52 ± 0.49 | 22.63 ± 0.89 | 6.46 ± 0.95 | 27.63 ± 1.12 | 4.84 ± 0.31 |
| 1 | Low | 60 | LL | 1.95 ± 0.23 | 100.51 ± 3.18 | 57.37 ± 2.50 | 2.95 ± 0.49 | 22.14 ± 0.89 | 26.48 ± 0.95 | 40.08 ± 1.12 | 4.96 ± 0.31 |
| 1 | Low | 60 | Bud | 3.67 ± 0.23 | 120.04 ± 3.18 | 34.20 ± 2.50 | 1.64 ± 0.49 | 18.46 ± 0.89 | 30.91 ± 0.95 | 48.32 ± 1.12 | 7.82 ± 0.31 |
| 1 | Low | 60 | Stem | 1.60 ± 0.23 | 31.84 ± 3.18 | 47.80 ± 2.50 | 4.23 ± 0.49 | 22.83 ± 0.89 | 6.86 ± 0.95 | 28.59 ± 1.12 | 3.58 ± 0.31 |
| 1 | High | 90 | LL | 1.93 ± 0.28 | 100.61 ± 3.90 | 56.66 ± 3.06 | 2.54 ± 0.59 | 23.37 ± 1.09 | 26.61 ± 1.17 | 40.37 ± 1.37 | 4.70 ± 0.37 |
| 1 | High | 90 | Bud | 3.09 ± 0.23 | 113.22 ± 3.18 | 34.08 ± 2.50 | 2.57 ± 0.49 | 18.51 ± 0.89 | 29.79 ± 0.95 | 45.80 ± 1.12 | 7.37 ± 0.31 |
| 1 | High | 90 | Stem | 1.66 ± 0.23 | 30.08 ± 3.18 | 48.22 ± 2.50 | 4.10 ± 0.49 | 22.99 ± 0.89 | 6.15 ± 0.95 | 28.23 ± 1.12 | 3.88 ± 0.31 |
| 7 | Low | 60 | LL | 1.76 ± 0.23 | 100.98 ± 3.18 | 53.91 ± 2.50 | 2.69 ± 0.49 | 22.73 ± 0.89 | 27.56 ± 0.95 | 44.01 ± 1.12 | 2.97 ± 0.31 |
| 7 | Low | 60 | Bud | 3.14 ± 0.23 | 117.49 ± 3.18 | 28.12 ± 2.50 | 2.62 ± 0.49 | 17.79 ± 0.89 | 30.82 ± 0.95 | 55.23 ± 1.12 | 3.45 ± 0.31 |
| 7 | Low | 60 | Stem | 2.05 ± 0.23 | 30.58 ± 3.18 | 48.18 ± 2.50 | 5.21 ± 0.49 | 23.24 ± 0.89 | 6.46 ± 0.95 | 32.31 ± 1.12 | 2.21 ± 0.31 |
| 7 | High | 90 | LL | 1.41 ± 0.23 | 98.11 ± 3.18 | 58.04 ± 2.50 | 2.74 ± 0.49 | 23.26 ± 0.89 | 27.38 ± 0.95 | 41.52 ± 1.12 | 3.17 ± 0.31 |
| 7 | High | 90 | Bud | 2.77 ± 0.23 | 117.51 ± 3.18 | 35.66 ± 2.50 | 2.04 ± 0.49 | 20.36 ± 0.89 | 32.10 ± 0.95 | 53.67 ± 1.12 | 4.01 ± 0.31 |
| 7 | High | 90 | Stem | 1.13 ± 0.23 | 28.81 ± 3.18 | 44.09 ± 2.50 | 5.15 ± 0.49 | 22.10 ± 0.89 | 6.03 ± 0.95 | 30.54 ± 1.12 | 1.86 ± 0.31 |
| 11 | Low | 60 | LL | 1.83 ± 0.23 | 105.82 ± 3.18 | 56.81 ± 2.50 | 3.03 ± 0.49 | 22.08 ± 0.89 | 27.79 ± 0.95 | 47.58 ± 1.12 | 1.94 ± 0.31 |
| 11 | Low | 60 | Bud | 2.68 ± 0.23 | 114.21 ± 3.18 | 29.84 ± 2.50 | 2.07 ± 0.49 | 18.80 ± 0.89 | 30.67 ± 0.95 | 57.84 ± 1.12 | 1.70 ± 0.31 |
| 11 | Low | 60 | Stem | 2.09 ± 0.23 | 32.94 ± 3.18 | 52.09 ± 2.50 | 4.14 ± 0.49 | 24.17 ± 0.89 | 6.53 ± 0.95 | 34.77 ± 1.12 | 1.79 ± 0.31 |
| 11 | High | 90 | LL | 1.36 ± 0.23 | 103.24 ± 3.18 | 55.09 ± 2.50 | 2.09 ± 0.49 | 18.99 ± 0.89 | 25.50 ± 0.95 | 45.92 ± 1.12 | 2.53 ± 0.31 |
| 11 | High | 90 | Bud | 2.02 ± 0.23 | 115.48 ± 3.18 | 28.62 ± 2.50 | 1.94 ± 0.49 | 18.18 ± 0.89 | 31.03 ± 0.95 | 56.49 ± 1.12 | 2.20 ± 0.31 |
| 11 | High | 90 | Stem | 1.04 ± 0.23 | 32.59 ± 3.18 | 45.67 ± 2.50 | 4.89 ± 0.49 | 21.88 ± 0.89 | 6.91 ± 0.95 | 32.54 ± 1.12 | 1.80 ± 0.31 |

**Table S2.** Spatial changes in gallic acid (GA), (+)-catechin, epicatechin (EC), epigallocatechin gallate (EGCG), epicatechin gallate (ECG), epigallocatechin (EGC), theobromine (TB) and caffeine (mg g^-1^ dry weight [DW]) in fresh tea shoots (large leaf [LL], bud and stem) from one variety of tea (Yabukita) during 11 days storage at 5^o^C and high [90 % glycerol] and low [60% glycerol] vapour pressure deficit (VPD). Data represent means (n = 3) ± standard deviation (SD). Interactions between main factors studied (storage time x VPD x spatial) were not significant (*p* >0.05).

| **Sampling day** | **VPD** | **Glycerol (%)** | **Spatial** | **GA** | **EGCG** | **EGC** | **(+)-catechin** | **EC** | **ECG** | **Caffeine** | **TB** |
| --- | --- | --- | --- | --- | --- | --- | --- | --- | --- | --- | --- |
| B1 | * | * | LL | 2.11 ± 0.17 | 60.02 ± 2.03 | 58.02 ± 2.74 | 0.78 ± 0.13 | 17.44 ± 0.67 | 10.15 ± 0.73 | 23.69 ± 1.11 | 0.40 ± 0.14 |
| B1 | * | * | Bud | 2.43 ± 0.17 | 68.89 ± 2.03 | 33.39 ± 2.74 | 1.06 ± 0.13 | 15.20 ± 0.67 | 16.48 ± 0.73 | 31.30 ± 1.11 | 2.01 ± 0.14 |
| B1 | * | * | Stem | 0.50 ± 0.17 | 12.42 ± 2.03 | 27.51 ± 2.74 | 2.88 ± 0.13 | 21.30 ± 0.67 | 2.96 ± 0.73 | 16.80 ± 1.11 | 0.46 ± 0.14 |
| B2 | * | * | LL | 1.39 ± 0.17 | 58.03 ± 2.03 | 61.09 ± 2.74 | 0.87 ± 0.13 | 17.17 ± 0.67 | 9.63 ± 0.73 | 23.41 ± 1.11 | 0.63 ± 0.14 |
| B2 | * | * | Bud | 2.96 ± 0.17 | 68.00 ± 2.03 | 37.09 ± 2.74 | 1.01 ± 0.13 | 14.58 ± 0.67 | 14.99 ± 0.73 | 30.31 ± 1.11 | 2.20 ± 0.14 |
| B2 | * | * | Stem | 0.77 ± 0.17 | 14.17 ± 2.03 | 30.77 ± 2.74 | 3.19 ± 0.13 | 19.31 ± 0.67 | 2.90 ± 0.73 | 17.63 ± 1.11 | 0.91 ± 0.14 |
| 1 | Low | 60 | LL | 2.09 ± 0.17 | 60.50 ± 2.03 | 63.24 ± 2.74 | 0.78 ± 0.13 | 17.35 ± 0.67 | 9.77 ± 0.73 | 23.69 ± 1.11 | 0.60 ± 0.14 |
| 1 | Low | 60 | Bud | 3.80 ± 0.17 | 73.14 ± 2.03 | 36.56 ± 2.74 | 0.94 ± 0.13 | 15.07 ± 0.67 | 16.37 ± 0.73 | 32.33 ± 1.11 | 1.74 ± 0.14 |
| 1 | Low | 60 | Stem | 0.76 ± 0.17 | 15.15 ± 2.03 | 31.95 ± 2.74 | 3.18 ± 0.13 | 21.61 ± 0.67 | 3.41 ± 0.73 | 18.27 ± 1.11 | 0.54 ± 0.14 |
| 1 | High | 90 | LL | 2.03 ± 0.17 | 61.59 ± 2.03 | 65.51 ± 2.74 | 0.88 ± 0.13 | 18.07 ± 0.67 | 10.49 ± 0.73 | 24.70 ± 1.11 | 0.73 ± 0.14 |
| 1 | High | 90 | Bud | 3.69 ± 0.17 | 73.99 ± 2.03 | 38.22 ± 2.74 | 1.15 ± 0.13 | 15.66 ± 0.67 | 16.52 ± 0.73 | 33.98 ± 1.11 | 1.82 ± 0.14 |
| 1 | High | 90 | Stem | 0.88 ± 0.17 | 14.74 ± 2.03 | 33.45 ± 2.74 | 3.24 ± 0.13 | 21.72 ± 0.67 | 3.10 ± 0.73 | 18.28 ± 1.11 | 0.62 ± 0.14 |
| 7 | Low | 60 | LL | 2.50 ± 0.17 | 63.57 ± 2.03 | 60.63 ± 2.74 | 1.00 ± 0.13 | 18.00 ± 0.67 | 10.68 ± 0.73 | 26.49 ± 1.11 | 0.37 ± 0.14 |
| 7 | Low | 60 | Bud | 3.02 ± 0.17 | 72.15 ± 2.03 | 35.87 ± 2.74 | 1.20 ± 0.13 | 15.87 ± 0.67 | 16.43 ± 0.73 | 36.18 ± 1.11 | 0.60 ± 0.14 |
| 7 | Low | 60 | Stem | 0.77 ± 0.17 | 13.58 ± 2.03 | 31.96 ± 2.74 | 3.21 ± 0.13 | 22.26 ± 0.67 | 3.04 ± 0.73 | 19.73 ± 1.11 | 0.17 ± 0.14 |
| 7 | High | 90 | LL | 2.29 ± 0.17 | 69.15 ± 2.03 | 59.10 ± 2.74 | 1.09 ± 0.13 | 19.00 ± 0.67 | 12.50 ± 0.73 | 29.19 ± 1.11 | 0.57 ± 0.14 |
| 7 | High | 90 | Bud | 3.47 ± 0.17 | 76.06 ± 2.03 | 34.47 ± 2.74 | 1.34 ± 0.13 | 15.77 ± 0.67 | 17.72 ± 0.73 | 38.80 ± 1.11 | 0.81 ± 0.14 |
| 7 | High | 90 | Stem | 0.76 ± 0.17 | 16.75 ± 2.03 | 35.21 ± 2.74 | 3.64 ± 0.13 | 22.70 ± 0.67 | 3.79 ± 0.73 | 22.03 ± 1.11 | 0.20 ± 0.14 |
| 11 | Low | 60 | LL | 2.09 ± 0.17 | 68.40 ± 2.03 | 65.02 ± 2.74 | 1.01 ± 0.13 | 19.41 ± 0.67 | 11.70 ± 0.73 | 28.97 ± 1.11 | 0.44 ± 0.14 |
| 11 | Low | 60 | Bud | 3.57 ± 0.17 | 72.26 ± 2.03 | 36.20 ± 2.74 | 1.23 ± 0.13 | 15.83 ± 0.67 | 16.21 ± 0.73 | 39.66 ± 1.11 | 0.54 ± 0.14 |
| 11 | Low | 60 | Stem | 0.71 ± 0.17 | 15.66 ± 2.03 | 34.32 ± 2.74 | 3.50 ± 0.13 | 23.02 ± 0.67 | 3.65 ± 0.73 | 21.64 ± 1.11 | 0.22 ± 0.14 |
| 11 | High | 90 | LL | 1.61 ± 0.17 | 67.17 ± 2.03 | 64.04 ± 2.74 | 1.02 ± 0.13 | 18.95 ± 0.67 | 12.11 ± 0.73 | 29.83 ± 1.11 | 0.48 ± 0.14 |
| 11 | High | 90 | Bud | 2.97 ± 0.17 | 73.81 ± 2.03 | 35.93 ± 2.74 | 1.18 ± 0.13 | 15.24 ± 0.67 | 16.68 ± 0.73 | 39.58 ± 1.11 | 0.53 ± 0.14 |
| 11 | High | 90 | Stem | 0.75 ± 0.17 | 17.65 ± 2.03 | 34.97 ± 2.74 | 3.49 ± 0.13 | 21.88 ± 0.67 | 3.86 ± 0.73 | 22.91 ± 1.11 | 0.29 ± 0.14 |

**Fig. S2.** Spatial changes in [a] epicatechin (EC) and [b] (+)-catechin (mg g^-1^ dry weight [DW]) in fresh tea shoots (large leaf [LL], bud and stem) from two varieties of tea (*viz.* Clone 2 and Yabukita) during 11 days storage at 5 ^°^C and high vapour pressure deficit (VPD). Data represent means (*n* = 3) ± standard deviation (SD).
